# Predictors of negative first SARS-CoV-2 RT-PCR despite final diagnosis of COVID-19 and association with outcome

**DOI:** 10.1038/s41598-021-82192-6

**Published:** 2021-01-27

**Authors:** Jean-Baptiste Lascarrou, Gwenhael Colin, Aurélie Le Thuaut, Nicolas Serck, Mickael Ohana, Bertrand Sauneuf, Guillaume Geri, Jean-Baptiste Mesland, Gaetane Ribeyre, Claire Hussenet, Anne Sophie Boureau, Thomas Gille

**Affiliations:** 1 Service de Médecine Intensive Réanimation, Centre Hospitalier Universitaire Hôtel-Dieu, 30 Bd. Jean Monnet, 44093 Nantes Cedex 1, France; 2 CRICS-TRIGGERSEP Network, Tours, France; 3 Médecine Intensive Réanimation, Centre Hospitalier Departemental, La Roche-sur-Yon, France; 4 Plateforme de Méthodologie et Biostatistique, CHU Nantes, 1 place Alexis Ricordeau, 44093 Nantes Cedex 9, France; 5 Unité de Soins Intensifs, Clinique Saint Pierre Ottignies, Ottignies, Belgium; 6 Service de Radiologie, CHRU Strasbourg, Strasbourg, France; 7 Réanimation - Médecine Intensive, Centre Hospitalier Public du Cotentin, BP208, 50102 Cherbourg-en-Cotentin, France; 8 Médecine Intensive Réanimation, CHU Ambroise Paré, Boulogne Billancourt, France; 9 Unité de Soins Intensifs, Hôpital de Jolimont, Jolimont, Belgium; 10 Centre Médical, Avignon, France; 11 Médecine Polyvalente, Nouvelles Cliniques Nantaises, Nantes, France; 12 Médecine Aigüe Gériatrique, CHU Nantes, Nantes, France; 13 Pneumologie, University Hospital Center Avicenne, AP-HP, Bobigny, France; 14 Inserm UMR 1272 “Hypoxia and the Lung”, Sorbonne Paris Nord University, Bobigny, France

**Keywords:** Medical research, Infectious diseases

## Abstract

Reverse transcriptase-polymerase chain reaction (RT-PCR) testing is an important tool for diagnosing coronavirus disease 2019 (COVID-19). However, performance concerns have emerged recently, notably regarding sensitivity. We hypothesized that the clinical, biological, and radiological characteristics of patients with a false-negative first RT-PCR test and a final diagnosis of COVID-19 might differ from those of patients with a positive first RT-PCR test. We conducted a multicenter matched case–control study in COVID-19 patients. Patients with a negative first RT-PCR test were matched to patients with a positive first RT-PCR test on age, sex, and initial admission unit (ward or intensive care). We included 80 cases and 80 controls between March 30, and June 22, 2020. Neither mortality at hospital discharge nor hospital stay length differed between the two groups (*P* = 0.80 and *P* = 0.54, respectively). By multivariate analysis, two factors were independently associated with a lower risk of a first false-negative test, namely, headache (adjusted OR [aOR], 0.07; 95% confidence interval [95% CI], 0.01–0.49]; *P* = 0.007) and fatigue/malaise (aOR, 0.16; 95% CI, 0.03–0.81; *P* = 0.027); two other factors were independently associated with a higher risk of a first false-negative test, namely, platelets > 207·10^3^ mm^−3^ (aOR, 3.81; 95% CI, 1.10–13.16]; *P* = 0.034) and C-reactive protein > 79.8 mg·L^−1^ (aOR, 4.00; 95% CI, 1.21–13.19; *P* = 0.023). Patients with suspected COVID-19 whose laboratory tests indicating marked inflammation were at higher risk of a first false-negative RT-PCR test. Strategies involving serial RT-PCR testing must be rigorously evaluated.

## Introduction

In December 2019, a novel coronavirus named severe acute respiratory syndrome coronavirus 2 (SARS-CoV-2) emerged in Wuhan City in China then spread rapidly throughout the world^1^. By September 1, 2020, more than 25,000,000 patients had been infected and 850 000 had died from coronavirus disease 2019 (COVID-19).

Coronaviruses are enveloped RNA viruses that are broadly distributed among humans, other mammals, and birds, causing respiratory, enteric, hepatic, and neurologic disorders. Many publications have highlighted the diversity of COVID-19 presentations, although respiratory symptoms are predominant^2^. The multiple and nonspecific symptoms of COVID 19 raise diagnostic challenges. A rapid and accurate diagnosis is essential to allow isolation, contact tracing, and the administration of treatments appropriate to the severity of the disease. SARS-CoV-2 was identified and sequenced by a Chinese team, who rapidly communicated their results, allowing clinicians worldwide to perform reverse transcriptase polymerase chain reaction (RT-PCR) testing on oropharyngeal or -nasopharyngeal swabs in patients with suspected COVID-19^2^.

Recently, however, concern has been raised about the performance of RT-PCR testing, notably regarding sensitivity. Two patients with false-negative RT-PCR tests were reported in South Korea in April 2020^3^. In a cohort of 219 confirmed COVID-19 patients matched to 205 patients with other causes of viral pneumonia, computed tomography (CT) outperformed nasopharyngeal RT-PCR testing to rule in or rule out COVID-19 disease^4^. While the analytic performance of SARS-CoV-2 RT-PCR testing has been well described^5^, its clinical performance may be diminished by several factors such as low levels of shedding^6^, sample collection site^7^, and technical proficiency of the sample collectors and handlers. Thus, patients who are ultimately proven to have COVID-19 may, particularly early in the course of their disease, have a negative RT-PCR test.

We hypothesized that the clinical and/or biological and/or radiological characteristics of patients with a false-negative first RT-PCR test but a final diagnosis of COVID-19 may differ from those of patients with a positive first RT-PCR test. We also hypothesized that outcomes might be better in patients with a false-negative first RT-PCR test than in those with a positive first RT-PCR test. To assess this hypothesis, we performed a case–control study among COVID-19 patients, in which patients with a negative first RT-PCR test were matched to patients with a positive first RT-PCR test.

## Materials and methods

### Study design and patients

This multicenter matched case–control study was conducted in patients admitted to 11 hospitals in France and Belgium. Cases were admitted patients who had a final diagnosis of COVID-19 despite a negative first RT-PCR test. Controls were patients from the same hospital who had a positive first RT-PCR test. For each case, 1 control was matched on sex, age, and initial admission unit (ward or intensive care).

The inclusion criteria were age older than 18 years and admission for an infectious condition with a final diagnosis of COVID-19. Non-inclusion criteria were pneumonia with biological identification of a causative agent other than SARS-CoV-2; pregnancy, recent delivery, or lactation; and adult under guardianship or curatorship.

### Outcomes

Our primary objective was to identify factors associated with a higher risk of a first false-negative RT-PCR test. We also assessed the treatments delivered, the need for and duration of mechanical ventilation, the occurrence of acute respiratory distress syndrome (ARDS), and vital status at hospital discharge.

### Data collection

At each participating center, the local investigator entered the study data into an electronic case report form (eCRF) (Castor EDC, Amsterdam, Netherlands). All data were anonymized, and no data could be traced back to the patient's identity. The following were collected: matching characteristics (age, sex, department of admission); baseline demographics and comorbidities; clinical and laboratory findings at hospital admission; history of the symptoms; radiological findings; RT-PCR test results (first RT-PCR test and, in the cases, whether the final RT-PCR test was positive); tests for other pathogens with the results; antiviral treatments; outcomes; and final diagnostic modalities for the cases.

### Ethics

The study was approved by the appropriate ethics committees (For France: Comité d’éthique de la Société de Réanimation de Langue Française, #20-26; and for Belgium: Comité d’Ethique 045 Clinique Saint Pierre), which waived the need for informed consent in keeping with legislation on retrospective analyses of anonymized data. All research was performed in accordance with relevant guidelines/regulations.

### Statistical analysis

The statistical analysis was performed according to STROBE guidelines^8^. Qualitative variables were described as number (%) and quantitative variables as mean ± SD if normally distributed and as median [25th–75th percentile] otherwise. Mortality at hospital discharge and hospitalization length were compared between cases and controls using conditional logistic regression to take into account paired data. Conditional logistic regression models were used to identify factors associated with a first negative RT-PCR test. Step-by-step backward selection was applied. Predefined factors associated with a first negative RT-PCR test at *P* values ≤ 0.2 by univariate analysis were then introduced into a multiple logistic regression model. Variables were kept if they were associated with *P* values ≤ 0.1 (conservative approach). The Homesher-Lemeshow test and visual inspection of residues were used to ensure the good quality of the regression. Quantitative variables were dichotomized according to their median. Selection of collinear variables was performed according to their clinical relevance. Model selection was based on the Akaike information criterion (AIC)^9^. The time from symptom onset to the first RT-PCR test was forced into all models, as it was found to be important in earlier studies. All statistical analyses were performed using SAS (Microsoft, Redmond, CA, USA).

### Sample size

Given the exploratory nature of our study, we did not estimate a sample size, but we aimed to include at least 50 cases and 50 controls (100 patients).

## Results

Between March 30 and June 22, 2020, we identified 82 cases. Among them, 2 were excluded because no matching control was found. We therefore analyzed 80 cases and 80 controls. Males predominated (66.3%), mean age was 64.1 ± 16.8 old, and most patients (71.3%) were admitted to wards.

### Cases

Among the 80 cases, 25 underwent chest radiography, whose findings were as follows: bilateral patchy opacities (n = 12), interstitial abnormalities (n = 7), ground-glass opacities (n = 4), local patchy opacities (n = 1), and normal (n = 1). A CT scan of the chest was obtained in 75 cases and usually showed ground-glass opacities (n = 69); interstitial abnormalities were seen in 4 patients, and the results were normal in 1 patient. No data were available for 1 patient.

Median time from symptom onset to RT-PCR testing was 6 [2.5–10.5] days in the cases and 5 [1.0–9.0] days in the controls (*P* = 0.27). In 11 cases, a subsequent RT-PCR test was performed, at a median of 11.0 [9.0–16.0] days after symptom onset, and gave a positive result.

The final diagnosis of COVID-19 in the 80 cases was based on one or more of the following: chest CT scan findings (n = 71), proven COVID-19 in a household member (n = 13), subsequent positive RT-PCR test on an oropharyngeal swab (n = 9), subsequent positive RT-PCR test on a tracheal aspirate sample (n = 4), subsequent positive RT-PCR test on sputum (n = 1), member of a known COVID-19 cluster (n = 2), and serological testing (n = 2).

### Cases and controls

Tables 1 and 2 detail the clinical and laboratory findings, respectively, in the cases and controls.

**Table 1:** Baseline characteristics of study population.

	Total N = 160	Cases N = 80	Controls N = 80	*P* value
**Matching characteristics**				
Age, mean ± SD	64.1 ± 16.8	64.0 ± 16.9	64.1 ± 16.7	–
Male, n (%)	106 (66.3%)	53 (66.3%)	53 (66.3%)	–
ICU admission, n (%)	46 (28.8%)	23 (28.8%)	23 (28.8%)	–
**Non-matching characteristics**				
Body mass index, median [IQR]	27.47 [24.45; 30.81]	27.31 [24.46; 29.09]	27.76 [23.57; 31.30]	0.28
Smoking history, n (%)	23 (14.7%)	13 (16.9%)	10 (12.7%)	0.47
Charlson index, median [IQR]	1 [0; 3]	1 [0; 2]	1 [0; 3]	0.96
Time from symptom onset to hospital admission, days, median [IQR]	7.00 [4.00; 11.00]	7.00 [4.00; 13.00]	7.00 [3.00; 10.00]	0.16
**Location**				
Belgium	22 (13.8%)	11 (13.8%)	11 (13.8%)	–
France	138 (86.3%)	69 (86.3%)	69 (86.3%)	
Time from symptom onset to ICU admission, days, median [IQR]	11 [7;14]	13 [7;15]	9 [7;13]	0.12
Temperature, °C, median [IQR]	37.7 [37.0;38.4]	37.5 [36.90;38.4]	38.0 [37.1;38.5]	0.11
Heart rate, beats/minute, median [IQR]	87 [75;102]	89 [80;105]	86 [74;99]	0.06
Respiratory rate, beats/minute, median [IQR]	25 [20;30]	24 [20;32]	25 [22;30]	0.77
Systolic blood pressure, mmHg, median [IQR]	132 [119;144]	130 [120;141]	136 [117.00;149]	0.48
Diastolic blood pressure, mmHg, median [IQR]	75 [66;84]	74 [65;82]	75 [67;84]	0.74
**Oxygen saturation on**				
Room air	101 (63.1%)	47 (58.8%)	54 (67.5%)	0.21
Oxygen therapy	59 (36.9%)	33 (41.3%)	26 (32.5%)	
History of fever, n (%)	127 (80.4%)	57 (72.2%)	70 (88.6%)	0.02
Oxygen saturation, %, median [IQR]	95 [93; 97]	94 [92; 97]	95 [93; 97]	0.98
Dry cough, n (%)	94 (59.5%)	45 (57.0%)	49 (62.0%)	0.47
Cough with bloody sputum, n (%)	31 (19.6%)	15 (19.0%)	16 (20.3%)	0.81
Sore throat, n (%)	10 (7.2%)	7 (10.0%)	3 (4.4%)	0.18
Rhinorrhea, n (%)	19 (13.1%)	10 (13.9%)	9 (12.3%)	0.79
Ear pain, n (%)	1 (0.7%)	0 (0.0%)	1 (1.4%)	0.99
Wheezing, n (%)	8 (5.3%)	6 (7.8%)	2 (2.7%)	0.42
Chest pain, n (%)	22 (14.6%)	13 (16.9%)	9 (12.2%)	0.37
Myalgia, n (%)	40 (27.8%)	14 (19.7%)	26 (35.6%)	0.024
Arthralgia, n (%)	7 (5.0%)	2 (2.9%)	5 (7.1%)	0.27
Fatigue/Malaise, n (%)	87 (56.9%)	38 (50.0%)	49 (63.6%)	0.04
Dyspnea, n (%)	107 (67.3%)	58 (73.4%)	49 (61.3%)	0.08
Lower chest wall indrawing, n (%)	11 (7.5%)	5 (6.9%)	6 (8.1%)	0.42
Headache, n (%)	22 (14.7)	7 (9.2%)	15 (20.3%)	0.04
Altered consciousness/confusion, n (%)	21 (13.9%)	9 (12.0%)	12 (15.8%)	0.33
Abdominal pain, n (%)	23 (15.1%)	11 (14.1%)	12 (16.2%)	0.59
Vomiting/Nausea, n (%)	25 (15.8%)	13 (16.5%)	12 (15.2%)	0.83
Diarrhea, n (%)	42 (26.9%)	19 (24.7%)	23 (29.1%)	0.55
Skin ulcers, n (%)	1 (0.7%)	1 (1.3%)	0 (0.0%)	0.99
Lymphadenopathy, n (%)	1 (0.7%)	1 (1.5%)	0 (0.0%)	0.99
Bleeding, n (%)	3 (2.0%)	2 (2.6%)	1 (1.3%)	0.57

*ICU* intensive care unit, *IQR* interquartile range.

**Table 2:** Laboratory test results in the study population.

	Total N = 160	Cases N = 80	Controls N = 80	*P* value
Hemoglobin at hospital admission (g/dL), median [IQR]	13.35 [12.00; 14.60]	13.35 [11.95; 14.55]	13.35 [12.20; 14.60]	0.86
White blood cells (10^3^ mm^−3^), median [IQR]	6.95 [5.23; 9.60]	8.67 [6.30; 11.30]	5.87 [4.80; 7.70]	0.004
Lymphocytes (cells·µL^−1^) , median [IQR]	1010 670; 1470]	1055 [750; 1460]	950 650; 1470]	0.34
Neutrophils (cells·µL^−1^) , median [IQR]	5.02 [3.50; 7.33]	4.67 [3.27; 7.30]	5.64 [3.75; 7.38]	0.63
Hematocrit (%), median [IQR]	39.6 [36.3; 43.0]	39.2 [36.1; 42.7]	39.9 [37.0; 43.0]	0.62
Platelets (10^3^ mm^−3^), median [IQR]	207.5 [156.5; 275.0]	244.0 [187.0; 330.0]	179.0 [147.0; 236.0]	0.0008
Prothrombin time (s), median [IQR]	14.00 [12.90; 15.40]	14.15 [13.35; 15.45]	13.60 [12.60; 15.40]	0.47
International normalized ratio (INR), median [IQR]	1.08 [1.00; 1.18]	1.08 [1.00; 1.20]	1.09 [0.98; 1.18]	0.52
Sodium (mEq.L^−1^), median [IQR]	137 135.0; 139.5]	136 [135.0; 139.0]	137 [135.0; 140.0]	0.85
Potassium (mEq·L^−1^), median [IQR]	4.1 [3.72; 4.30]	4.1 [3.79; 4.40]	4.0 [3.70; 4.30]	0.52
Glucose (mmol·L^−1^), median [IQR]	6.31 [5.75; 7.63]	6.50 [5.80; 7.50]	6.30 [5.50; 7.90]	0.50
Blood urea nitrogen (mmol·L^−1^), median [IQR]	7.0 [4.70; 11.42]	7.3 [5.10; 11.00]	6.7 [4.30; 12.10]	0.43
Creatinine (µmol·L^−1^), median [IQR]	84.0 [67.0; 104.0]	84.5 [68.0; 104.0]	83.0 [66.0; 104.0]	0.35
Alanine aminotransferase (U·L^−1^), median [IQR]	34.0 [25.0; 53.0]	29.4 [21.0; 46.0]	39.0 [31.5; 59.0]	0.024
Aspartate aminotransferase (U·L^-1^), median [IQR]	47.0 [32.0; 70.0]	40.0 [26.8; 66.0]	54.7 [36.9; 77.50]	0.02
Total bilirubin (µmol·L^−1^), median [IQR]	9.00 [6.00; 12.00]	8.78 [6.00; 14.00]	9.00 [6.00; 11.97]	0.41
Lactate (mmol·L^−1^), median [IQR]	1.3 [0.9; 1.7]	1.3 [0.9; 1.9]	1.2 [0.9; 1.5]	0.37
Procalcitonin (ng·mL^−1^), median [IQR]	0.19 [0.08; 0.49]	0.18 [0.11; 0.28]	0.21 [0.08; 0.89]	0.38
C-reactive protein (mg·L^−1^), median [IQR]	79.8 [40.0; 179.0]	103.6 [42.0; 214.0]	63.5 [36.6; 131.0]	0.14

*IQR* interquartile range.

The proportion of patients who received at least one treatment (chloroquine, corticosteroids, lopinavir/ritonavir, macrolides, and/or tocilizumab) was not significantly different between the cases and the controls (*P* = 0.26) (Table 3). Mechanical ventilation was required for 10 (12.7%) cases and 14 (17.7%) controls (*P* = 0.177), for a duration of 21 [16–35] days in the cases and 15 [5–21] days in the controls (*P* = 0.197).

**Table 3:** Treatments and outcomes.

	Total N = 160	Cases N = 80	Controls N = 80	*P* value
Lopinavir/ritonavir, n (%)	17 (10.6%)	5 (6.3%)	12 (15.0%)	0.06
Remdesivir, n (%)	0	0	0	–
Hydroxychloroquine, n (%)	39 (24.5%)	19 (23.8%)	20 (25.3%)	0.78
Macrolides, n (%)	70 (43.8%)	34 (42.5%)	36 (45.0%)	0.68
Corticosteroids, n (%)	10 (6.3%)	6 (7.5%)	4 (5.0%)	0.53
Tocilizumab, n (%)	3 (1.9%)	1 (1.3%)	2 (2.5%)	0.57
At least one treatment, n (%)	62 (38.8%)	28 (35.0%)	34 (42.5%)	0.26
**Outcome at hospital discharge**				
Discharged alive	135 (85.4%)	68 (86.1%)	67 (84.8%)	0.80
Died	23 (14.6%)	11 (13.9%)	12 (15.2%)	
Time from hospital admission to death, days, median [IQR]	9.0 [5.0; 17.0]	10.0 [6.0; 23.0]	6.5 [4.5; 16.5]	0.66
Hospital stay length, days, median [IQR]	8.0 [4.0; 15.0]	8.0 [4.5; 16.0]	8.5 [4.0; 15.0]	0.72
Time from hospital admission to hospital discharge or death, days, median [IQR]	8.0 [5.0; 16.0]	8.0 [5.0; 17.0]	8.0 [4.0; 16.0]	0.54
Mechanical ventilation, n (%)	24 (15.2%)	10 (12.7%)	14 (17.7%)	0.17
Duration of mechanical ventilation, days, median [IQR]	18.0 [11.0; 27.0]	21.5 [16.0; 35.0]	15.5 [5.0; 21.0]	0.19
ARDS, n (%)	29 (18.7%)	14 (18.4%)	15 (19; 0%)	0.59
**Grade of ARDS, n (%)**				
Mild	2 (6.9%)	2 (14.3%)	0	0.65
Moderate	9 (31.0%)	3 (21.4%)	6 (40.0%)	
Severe	18 (62.1%)	9 (64.3%)	9 (60.0%)	

*IQR* interquartile range, *ARDS* acute respiratory distress syndrome.

Neither hospital mortality nor hospital stay length differed significantly between the cases and controls (*P* = 0.80 and *P* = 0.54, respectively).

### Risk factors for a first false-negative RT-PCR test

By univariate analysis, factors associated with a lower risk of a first false-positive test were fatigue/malaise (*P* = 0.048), headache (*P* = 0.048), history of fever (*P* = 0.020), myalgia (0.024), and liver enzyme elevation (alanine aminotransferase (ALAT) and aspartate aminotransferase (ASAT), *P* = 0.024 for both). Factors associated with a higher risk of a first false-negative RT-PCR test were a platelet count above 207/10^3^·mm^−3^ (*P* = 0.002), a white blood cell count above 6.95/10^3^·mm^−3^ (*P* = 0.0003).

**Figure 1:**
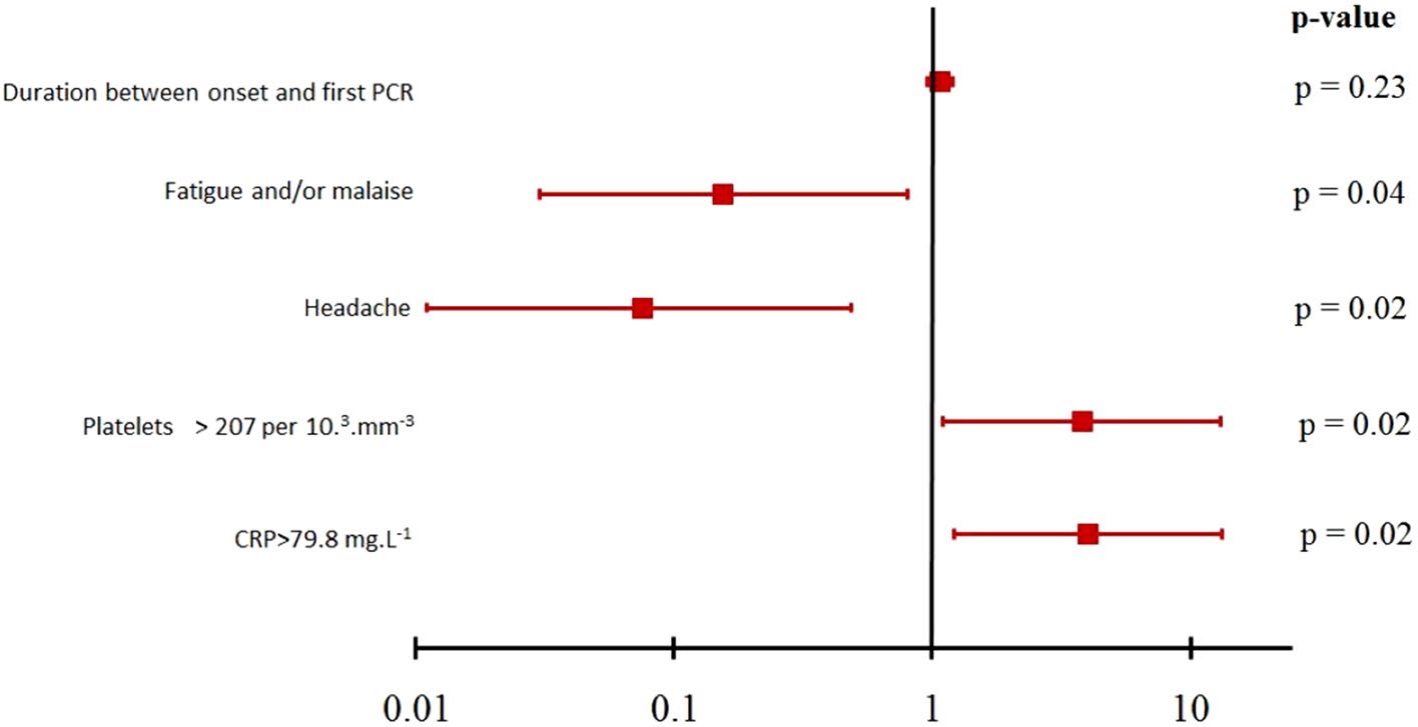
Forrest plot of multivariable analysis of factors associated with a first false-negative SARS-CoV-2 RT-PCR test.

Because ASAT and ALAT were collinear with the platelet count, and the white blood cell count was collinear with the CRP level, these variables were not included in the multivariate analysis. Figure 1 reports the result of multivariate analysis (AIC: 54.8 and BIC: 69.1).

## Discussion

In this study, patients with high platelet counts and/or CRP levels were at increased risk of having a false-negative first RT-PCR test. In contrast, patients with non-specific symptoms such as fatigue/malaise, headache, fever, and/or myalgia less often had a false-negative first RT-PCR. The time from symptom onset and RT-PCR testing was not associated with a false-negative result. Finally, patients with a first false-negative test did not differ from those with a first positive test regarding the treatments received, need for mechanical ventilation, or hospital mortality.

Three main conclusions can be drawn from our findings. First, time from symptom onset to RT-PCR testing was not associated with test positivity. This result is probably ascribable in part to difficulties in obtaining an accurate history of the course of the symptoms. Older patients often have difficulty timing their symptoms, as has been described for myocardial infarction^10^. Additionally, in one study up to 25% of older patients experienced delirium when ill with COVID-19^11^. The mean age of our patients was 64 ± 17 years. Second, the association between a high CRP level and a greater risk of a first false-negative RT-PCR test is of interest, because it is consistent with the major role for the cytokine storm in severe or fatal COVID-19, regardless of viral load^12^. In the RECOVERY trial, corticosteroids were the only treatment proven to be effective in reducing mortality in patients with COVID-19^13^. Only a small proportion of our patients received corticosteroids, but our study took place before evidence of beneficial effects of an early short course of corticosteroids was published. Additional data suggest that corticosteroids have the greatest benefits in patients whose CRP levels are above 20 mg/dL^14^. A positive RT-PCR test was not required for inclusion in the RECOVERY trial (11% of the whole cohort). Interestingly, in one study headache was associated with intermittently negative RT-PCR tests in patients with COVID-19^15^, whereas in our study headache was independently associated with a lower risk of a first false-negative RT-PCR test. Third, in the cases, the final diagnosis of COVID-19 was usually made by CT scan and not by RT-PCR testing. It has been suggested that only patients with a positive RT-PCR test should be included in clinical trials^16^. Conceivably, physicians may be less likely to prescribe the treatments used for COVID-19 to patients with negative RT-PCR results. The overall sensitivity of RT-PCR testing has been reported to be 70%^17^. However, the time from symptom onset to RT-PCR testing has a major impact on sensitivity. Thus, the false-negative rate fell from 38% on the first day with symptoms to 20% on day 8 then increased again^18^. In a study involving serial testing, 19 patients with a high probability of COVID-19 repeatedly had negative RT-PCR tests^19^. The usefulness of repeating RT-PCR tests over time in patients with initially negative results but a high suspicion of COVID-19 deserves to be evaluated. In a study from China, CT scan was more sensitive than RT-PCR for diagnosing COVID-19 and was deemed useful as the primary tool for detecting cases of COVID-19 during an epidemic^20^ but in a context of high probability of COVID-19 before CT scan. A strategy of large CT scan use for screening deserve evaluation: CT scan may have a place in diagnosis when RT-PCR is not available or when the results are not available and the diagnosis of SARS-CoV-2 infection changes management^21^.

Our study took place during the first epidemic wave in France and Belgium and included only patients requiring hospitalization. Therefore, pre-test probability of COVID-19 was high. We carefully selected hospitalized patients with several strong arguments for COVID-19 and a final diagnosis of COVID-19 at hospital discharge.

Some limitations of our study must be highlighted. First, negative RT-PCR testing can indicate infection by other agents. However, 45 (59.96%) cases also tested negative for other pathogens during their hospital stay, and the final diagnosis of COVID-19 was established based on all the available data, including the chest CT results, which were typical in 88.75% of cases. Second, imperfect collection of the sample for RT-PCR can result in false-negatives. However, all samples were collected in hospitals by trained nurses who used a dedicated protocol. Third, our sample size was limited, but we chose to include only cases with robust arguments for COVID-19, notably obtained by chest CT, whose availability was limited during the first pandemic wave in Europe. Last, we included patients from several centers that used different RT-PCR detection kits. However, evidence suggests similar performance of all available RT-PCR kits^22,23^.

## Conclusions

Patients with a first negative RT-PCR test for COVID-19 had higher inflammation markers, even at a median duration of 6 days after symptom onset, compared to patients with a positive first test. Decisions to use treatments such as corticosteroids known to be effective in COVID-19 cannot be based only on the RT-PCR test results. In patients with suspected COVID-19, the diagnosis must rest not only on RT-PCR test results but also on the clinical presentation and on the findings from other tests, most notably chest CT. Strategies involving serial RT-PCR testing and large use of CT scan for diagnosis must be rigorously evaluated.

## Data Availability

The datasets generated during and/or analysed during the current study are available from the corresponding author on reasonable request.
